# Orbitocranial resection for treatment of lacrimal gland malignant tumors with intracranial extension

**DOI:** 10.1016/j.bjorl.2026.101921

**Published:** 2026-10-14

**Authors:** Ying Xia, Yan Sui, Guo-Chao Zhang, Si-Yi Yang, Wen-Fu Lu, Ping-An Wu

**Affiliations:** a Division of Head and Neck Surgery, Department of Surgery, The University of Hong Kong-Shenzhen Hospital, Shenzhen, Guangdong, P.R. China; b School of Medicine, Shenzhen University, Shenzhen, Guangdong, P.R. China

**Keywords:** Lacrimal gland carcinoma, Intracranial extension, Orbitocranial approach, Skull base surgery, Orbital exenteration

## Abstract

**Objectives:**

This study reports our experience with multidisciplinary surgical management of lacrimal gland malignant tumors with intracranial extension using the orbitocranial approach.

**Methods:**

We reviewed treatment strategies and surgical approaches for lacrimal gland malignant tumors with intracranial extension and present 2 illustrative patients who underwent orbitocranial resection at our institution in 2015. Clinical records, radiographic images, histologic sections, and archived pathology specimens were reviewed.

**Results:**

Both patients underwent orbitocranial tumor resection, followed by postoperative chemoradiotherapy. Orbital reconstruction was performed using a temporalis fascia flap and split calvarial bone grafts, with split-thickness skin grafting to line the orbital cavity. The follow-up periods were 69- and 66-months, respectively. Both patients remained alive without evidence of disease recurrence during follow-up.

**Conclusions:**

The orbitocranial approach, combined with postoperative chemoradiotherapy, may provide satisfactory local and regional control in selected patients with lacrimal gland malignant tumors with intracranial extension. Long-term surveillance remains important because patients may remain at risk for disease recurrence or distant metastasis.

## Highlights

• Orbitocranial resection provides surgical access for selected lacrimal gland malignancies with intracranial extension.
• The approach allows en bloc tumor resection and skull-base reconstruction in selected patients.
• Two institutional cases illustrate surgical technique and long-term follow-up outcomes.
• This report summarizes current evidence and institutional experience in managing these rare tumors.

## Introduction

Lacrimal gland lesions are relatively rare, with an estimated incidence of 1.3/1,000,000 year.^1^ Malignant lacrimal gland tumors are even rarer, accounting for approximately one-quarter of all lacrimal gland lesions. The most common malignant lacrimal tumors are lymphomas (38%–58%), followed by adenoid cystic carcinomas (13%–30%), carcinomas ex pleomorphic adenomas (8%–10%), adenocarcinomas (3%–10%), and mucoepidermoid carcinomas (2%–5%). Cases of locally advanced lacrimal malignancies involving the anterior skull base and middle cranial fossa are seldom reported.^1^^,^^2^ Generally, patients with locally advanced malignant tumors of the lacrimal gland have a poor prognosis, and optimal treatment strategies remain difficult to establish.

The optimal treatment for locally advanced malignant lacrimal gland tumors remains controversial. Some authors have reported favorable outcomes with conservative globe-sparing surgical treatments followed by adjuvant radiotherapy, with or without systemic therapy.^3^, ^4^, ^5^, ^6^ Others have suggested that radical exenteration with or without removal of the surrounding bone may be considered in selected patients, particularly when intracranial extension is present.^7^, ^8^, ^9^ In recent years, patients at our center with locally advanced malignant lacrimal tumors involving the anterior and middle cranial fossae have undergone radical exenteration via the orbitocranial approach combined with postoperative chemoradiotherapy.^10^ This approach provides wide, multidirectional access to the anterior and middle cranial fossae and may facilitate resection of tumors involving the orbital apex and skull base. The aim of this study is to describe our surgical experience, treatment outcomes, and technical considerations associated with the orbitocranial approach for lacrimal gland malignant tumors with intracranial extension.

## Invasive characteristics and comprehensive treatment strategies of lacrimal gland cancer

Lacrimal gland cancer often invades and destroys the orbital bone and the anterior and middle cranial fossae. Additionally, distant metastases are not uncommon. The treatment of lacrimal gland cancer typically involves surgery with or without adjuvant radiotherapy and systemic therapy.^11^ Studies by Tse et al.^12^ and Ma et al.^13^ have highlighted the role of chemotherapy as part of multidisciplinary management for patients with Lacrimal Gland Adenoid Cystic Carcinoma (LGACC). Additionally, research by Anjum et al.^14^ indicates that Vascular Endothelial Growth Factor (VEGF) and Hypoxia-Inducible Factor 1ɑ (HIF-1ɑ) play significant roles in the pathogenesis and aggressive behavior of LGACC, making them potential therapeutic targets for LGACC.

## Epidemiology and recurrence risks of common malignant lacrimal gland tumors

Adenoid cystic carcinoma is the most common malignant epithelial tumor, accounting for 20%–30% of all malignant lacrimal gland tumors.^11^^,^^15^ An examination of 118 LGACC cases recorded between 1999 and 2016 in the National Cancer Institute’s Surveillance, Epidemiology, and End Results (SEER) database showed that the majority of diagnoses occurred between the ages of 40 and 69, with the median age falling within the range of 50 to 59-years. Males represented 63.6% of the cases, and the left-to-right eye incidence ratio was 1.03:1, with age being a key prognostic factor for LGACC (worse survival in patients ≥60-years).^15^ Carcinoma ex pleomorphic adenoma is another important malignant epithelial tumor of the lacrimal gland, accounting for approximately 8–10% of malignant lacrimal gland tumors.^11^^,^^16^ These tumors typically arise from pre-existing pleomorphic adenomas and may demonstrate aggressive local behavior following malignant transformation. Complete surgical excision remains the cornerstone of treatment for achieving optimal local disease control.^16^

## Mechanisms and pathways of intracranial invasion

Malignant lacrimal gland tumors have a greater likelihood of intracranial invasion due to several factors: the presence of numerous neural and vascular structures in the orbit; the direct connection between the orbital bones and the intracranial cavity; and the close association between the periorbit and the nerve sheath.^17^^,^^18^ These factors increase the risk of intracranial invasion via perineural, vascular, intraosseous, and meningeal routes.

## Surgical management and outcomes

Locally advanced malignant tumors of the lacrimal gland are known for their high recurrence rates despite surgical treatment, radiotherapy, and chemotherapy. The optimal local therapy for these tumors remains controversial. Some studies support eye-sparing surgery combined with adjuvant radiotherapy,^3^, ^4^, ^5^, ^6^ whereas others advocate more radical surgical approaches for selected patients with advanced disease.^7^^,^^9^ In the study by Han et al.,^5^ the long-term outcomes of 10 patients with LGACC treated with eye-sparing surgery combined with adjuvant radiotherapy were evaluated. With a median follow-up of 89.5-months, no systemic metastases were reported. The results demonstrated that this treatment approach provides significant local control and survival benefits for patients with orbit-confined LGACC. Rapidis et al.^9^ discussed the surgical management of malignant orbital and orbitomaxillary tumors and emphasized that treatment should be individualized according to tumor histology, extent, and surgical feasibility. However, the survival benefit of extensive cranio-orbital resection remains uncertain, and Rose et al.^19^ reported no clear survival advantage associated with this approach. In summary, radical surgery may be considered in selected patients with moderate to advanced lacrimal gland malignancies.

## Skull base surgical approaches

The frontotemporosphenoidal craniotomy provides access to many structures within the anterior, middle, and interpeduncular fossae.^20^ Nevertheless, the associated surgical corridor is both long and narrow. To improve exposure without excessive brain retraction, various skull base procedures have been developed to disarticulate the orbit and zygoma. As early as 1981, Marsh et al.^21^ proposed a combined intracranial and extracranial radical resection approach for LGACC, accompanied by treatment recommendations based on an understanding of the disease's natural history, regional anatomy, and contemporary therapeutic literature. They emphasized that the feasibility of adequate tumor ablation depended on proper evaluation, including Computed Tomography (CT) imaging, an initial exploratory craniotomy, and intraoperative frozen section analysis of the cranial nerves traversing the orbit. Once resectability was confirmed, an 'en bloc' curative intracranial and extracranial resection was performed, encompassing the tumor, lacrimal gland, and all adjacent structures. For anticipated adjuvant radiotherapy, the defect was immediately reconstructed using an “ice-cream cone” forehead flap, and orbital prosthesis installation was planned immediately after radiation effects subsided. Postoperative CT imaging served as a baseline for follow-up. These recommendations remain relevant today as a reference for cranio-orbital approaches in managing LGACC. The surgical approach of orbital exenteration combined with resection of tumors with intracranial extension via a fronto-orbito-zygomatic approach in conjunction with a transfacial approach has also been reported.^18^

Among the related skull base approaches, the orbitozygomatic osteotomy is perhaps the best-known and most widely used, supported by quantitative measurements that document an increased field-of-view angle. However, findings from a recent study using human cadavers suggest that removal of the lateral orbital rim alone may be sufficient to reach many of the same targets for which the orbitozygomatic craniotomy has traditionally been used. Inclusion of the zygomatic arch in the osteotomy did not significantly enhance surgical exposure.^22^ In this study, using an orbitocranial approach, the lateral orbital rim osteotomy was performed in conjunction with a standard frontotemporosphenoidal craniotomy and substituted for the orbitozygomatic craniotomy to treat specific lesions.

In each case, disarticulation of the superior and lateral orbital bones as a surgical margin provided a satisfactory corridor for accessing these lesions. If necessary, the bony orbital rim can be reconstructed with split calvarial bone grafts or with an osseous component from a free tissue transfer. This procedure ensures an adequate surgical margin without causing significant functional or aesthetic impairment compared to exenteration alone.

Compared with the orbitozygomatic osteotomy, the orbitocranial approach has several advantages. Technically, it is simpler, allowing for a quicker operation with a lower risk of cosmetic deformities. Malocclusion is rare after either procedure. Muscle-induced malocclusion related to masseter or temporalis muscle spasm is less likely after the orbitocranial approach with lateral orbital rim osteotomy, due to reduced manipulation of jaw muscles.

## Novel surgical approach for lacrimal gland malignant tumors with intracranial extension: our experience

Our institution has adopted a multidisciplinary orbitocranial approach for the management of lacrimal gland malignant tumors with intracranial extension, combining radical tumor resection with orbital and skull-base reconstruction. This strategy integrates expertise from head and neck surgeons and neurosurgeons and aims to achieve adequate surgical margins while minimizing morbidity.

The surgical procedure begins with rotation of the head approximately 45 degrees to the contralateral side and a coronal incision to expose the orbit, superior and lateral orbital rims, and zygoma. The scalp is elevated anteriorly, and the temporalis muscle is reflected to allow a frontotemporal craniotomy. Following careful dissection of the dura from the orbital roof with minimal brain retraction, osteotomies of the superior and lateral orbital rims are performed to improve multidirectional access to the orbital apex and skull base.

The superior osteotomy extends posteriorly toward the orbital apex. The lateral osteotomy traverses the zygomatic and sphenoid portions of the lateral orbital wall into the sphenoid wing. This allows lateral mobilization of the orbital wall and facilitates exposure of lesions involving the orbital apex and adjacent skull base. When required, the orbital contents, involved dura, and affected orbital walls are removed en bloc.

Reconstruction is performed using temporalis fascia repair, split calvarial bone graft reconstruction harvested from the craniotomy flap, titanium miniplate fixation, and split-thickness skin grafting when appropriate. Long-term MRI surveillance is performed postoperatively to facilitate early detection of local recurrence.

## Case report

### Patients and evaluation

We retrospectively reviewed two patients with lacrimal gland malignant tumors involving intracranial extension who underwent orbitocranial resection at our institution in 2015. Preoperative evaluation included ophthalmologic examination, Computed Tomography (CT), and Magnetic Resonance Imaging (MRI) to assess orbital, skull-base, and dural involvement (Fig. 1, Fig. 2, Fig. 3, Fig. 4). Histopathological diagnoses were confirmed based on archived pathology reports, representative histopathologic slides, and multidisciplinary review. This retrospective study was approved by the Ethics Committee of The University of Hong Kong-Shenzhen Hospital (Approval n° 16−4.伦[2019]049-003), and written informed consent for publication was obtained from both patients.

**Fig. 1:**
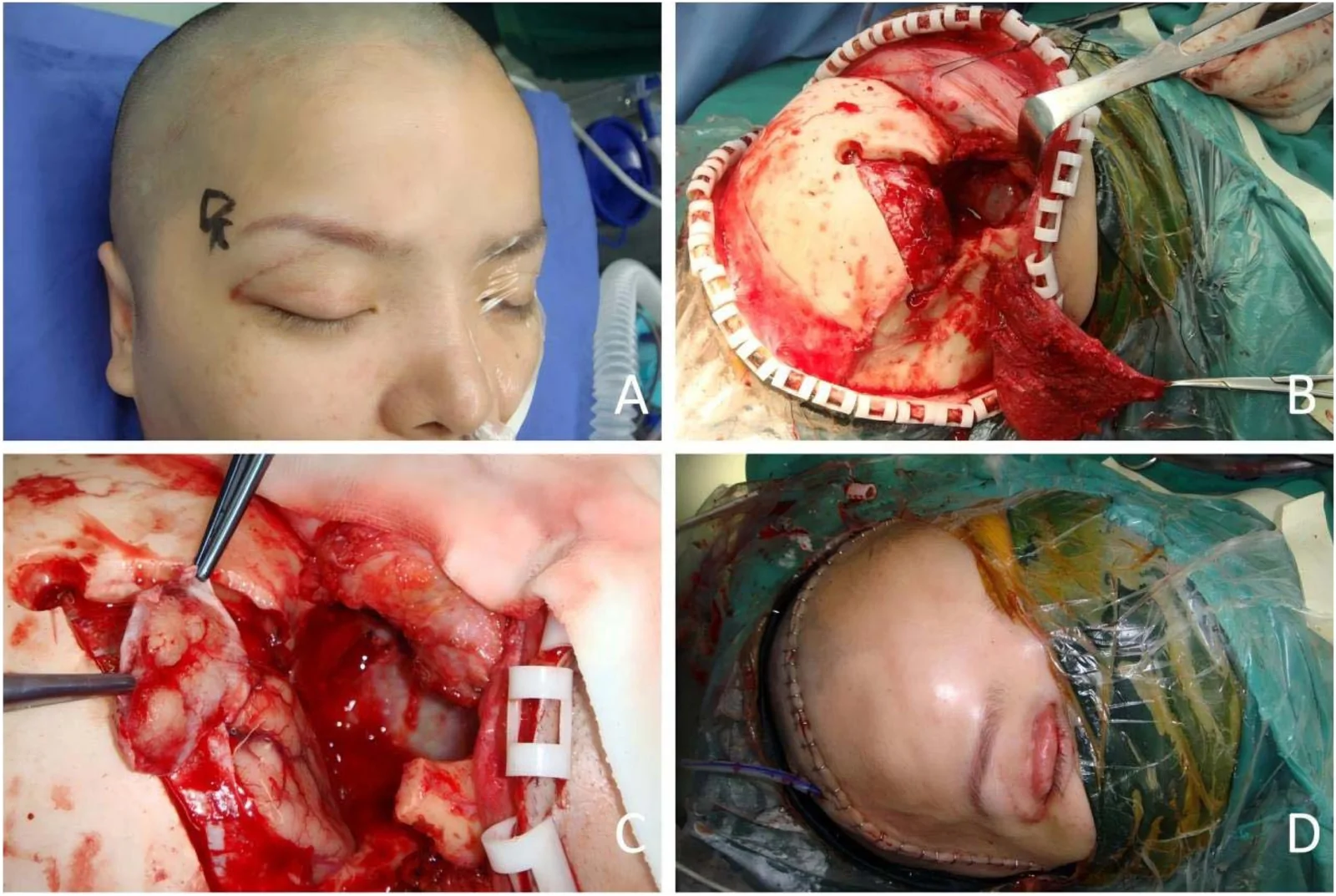
Case 1: Intraoperative photographs of the orbitocranial approach. (A) Preoperative appearance. (B) Orbitocranial approach with superior and lateral orbital rim osteotomy. (C) Intracranial tumor involvement following exposure of the anterior cranial fossa. (D) Immediate postoperative appearance.

**Fig. 2:**
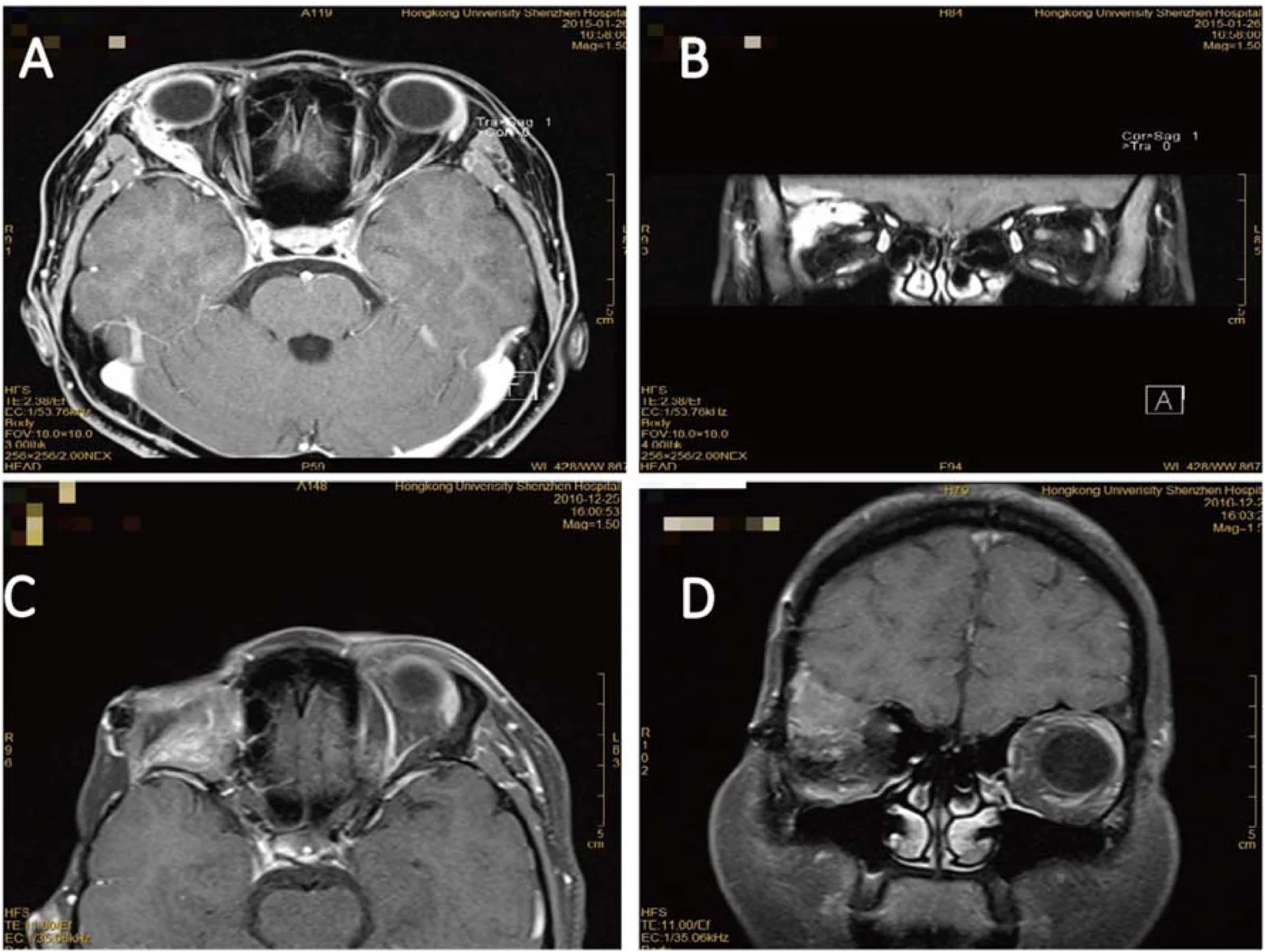
Case 1: Preoperative and postoperative Magnetic Resonance Imaging (MRI). Preoperative axial (A) and coronal (B) gadolinium-enhanced T1-weighted MRI demonstrating a lacrimal gland malignant tumor involving the orbit with extension into the anterior skull base and middle cranial fossa. Postoperative axial (C) and coronal (D) gadolinium-enhanced T1-weighted MRI obtained 2-years after surgery demonstrating complete tumor removal and stable reconstruction of the orbital cavity using a temporalis fascia flap.

**Fig. 3:**
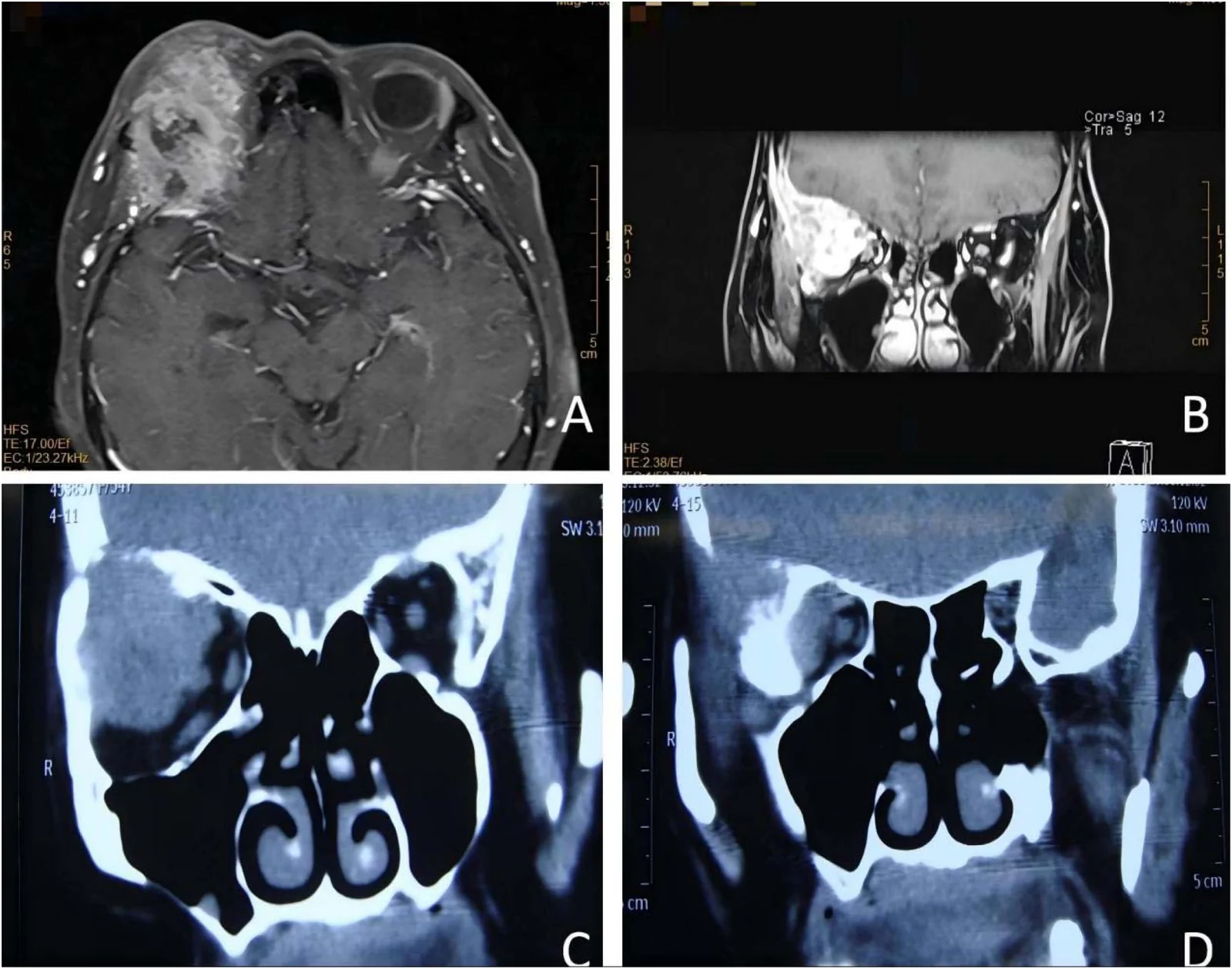
Case 2 Preoperative imaging studies. Axial (A) and coronal (B) gadolinium-enhanced T1-weighted MRI demonstrating a lacrimal gland malignant tumor involving the orbit. Coronal Computed Tomography (CT) images (C–D) demonstrating extension into the anterior and middle cranial fossae with associated orbital wall destruction and medial displacement of the globe.

**Fig. 4:**
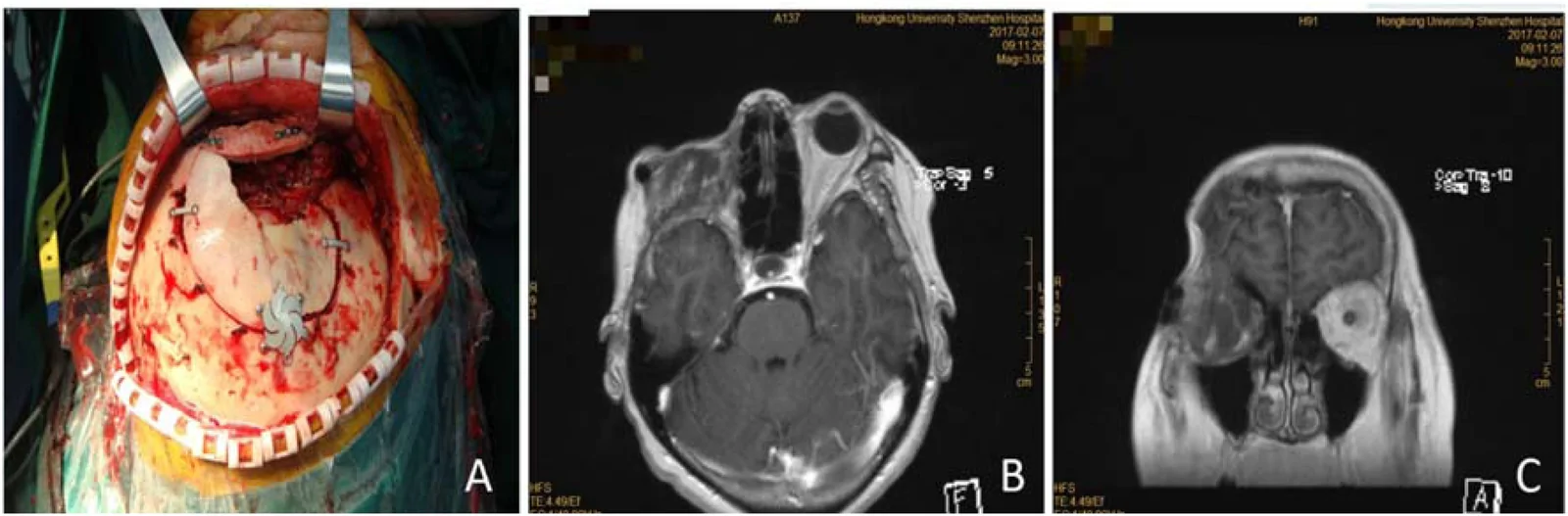
Case 2: Intraoperative and postoperative findings. (A) Intraoperative photograph demonstrating orbital wall reconstruction using split calvarial bone grafts harvested from the craniotomy flap. Postoperative axial (B) and Coronal (C) gadolinium-enhanced T1-weighted MRI demonstrating complete tumor removal and stable orbital reconstruction using a temporalis fascia flap.

### Surgical technique

The surgical procedure was performed using the orbitocranial approach described above. The orbital contents, involved dura, and affected orbital walls were removed en bloc when necessary. Reconstruction was performed using temporalis fascia repair, split calvarial bone graft reconstruction, titanium fixation, and split-thickness skin grafting as appropriate. Postoperative adjuvant therapy was administered according to histopathologic findings, margin status, and skull-base involvement.

## Case 1

A 33-year-old woman presented with progressive right orbital swelling and proptosis. Preoperative MRI demonstrated a lacrimal gland malignant tumor with extension into the anterior skull base and middle cranial fossa (Fig. 1, Fig. 2). The patient underwent orbitocranial tumor resection with en bloc removal of the orbital contents, involved dura, and affected orbital walls. Orbital reconstruction was performed using a temporalis fascia flap and split calvarial bone graft reconstruction. Histopathologic examination confirmed adenoid cystic carcinoma with dural/skull-base involvement (Fig. 5A–B). Final histopathologic examination confirmed negative surgical margins. Postoperative adjuvant chemoradiotherapy consisting of 60 Gy in 30 fractions with cisplatin-based chemotherapy was administered. No cerebrospinal fluid leakage, wound infection, flap necrosis, or new neurological deficit occurred postoperatively. At 69-months of follow-up with serial MRI surveillance, no evidence of disease recurrence was identified.

**Fig. 5:**
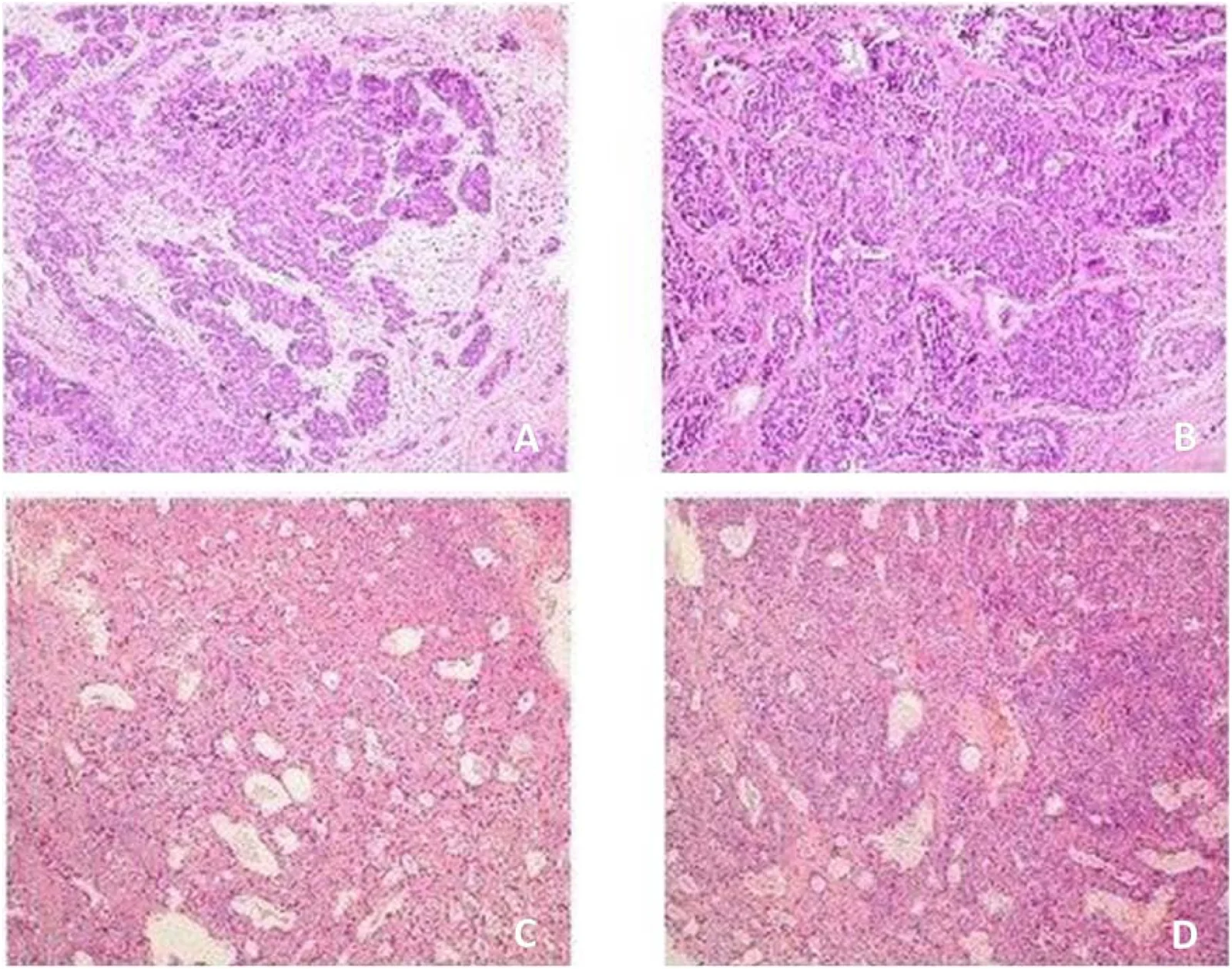
Histopathologic findings. (A–B) Case 1: Adenoid cystic carcinoma with dural and skull-base involvement. (C–D) Case 2: Carcinoma ex pleomorphic adenoma of the lacrimal gland. Representative hematoxylin and eosin–stained sections are shown.

## Case 2

A 55-year-old woman presented with progressive orbital pain and visual impairment. Imaging studies demonstrated a lacrimal gland malignant tumor involving the orbital apex with intracranial extension to the anterior and middle cranial fossae (Fig. 3, Fig. 4). The patient underwent orbitocranial resection with removal of the involved orbital walls and affected dura. Reconstruction was performed using split calvarial bone graft reconstruction and temporalis fascia repair. Histopathologic examination confirmed carcinoma ex pleomorphic adenoma of the lacrimal gland (Fig. 5C–D). Final histopathologic examination confirmed negative surgical margins. Postoperative cisplatin-based chemoradiotherapy was administered. Postoperative recovery was uneventful without cerebrospinal fluid leakage, wound complications, or flap failure. At 66-months of follow-up with clinical examination and serial MRI surveillance, the patient remained alive without evidence of disease recurrence.

### Outcomes

Both patients underwent successful orbitocranial tumor resection followed by postoperative adjuvant therapy. Long-term follow-up with serial MRI surveillance demonstrated no evidence of disease recurrence during follow-up. No major perioperative complications, cerebrospinal fluid leakage, wound infection, flap necrosis, or additional neurological deficits occurred in either patient. Both patients remained alive without evidence of disease during follow-up.

## Challenges and limitations in lacrimal gland tumor treatment

Due to the extreme rarity of lacrimal gland tumors with intracranial extension, large clinical series are difficult to obtain. Therefore, detailed descriptions of surgical techniques and institutional experience remain valuable for guiding clinicians managing similar cases. This study is limited by its retrospective design and the inclusion of only two patients. Although postoperative adjuvant therapy was administered in our patients, the role of systemic chemotherapy for locally advanced lacrimal gland malignancies remains uncertain and requires further investigation (Table 1).

**Table 1:** Clinical characteristics, treatment, and outcomes of the two patients with lacrimal gland malignant tumors with intracranial extension.

Variable	Case 1	Case 2
Age/Sex	33/Female	55/Female
Presenting symptoms	Progressive right orbital swelling and proptosis	Progressive orbital pain and visual impairment
Histopathology	Adenoid cystic carcinoma	Carcinoma ex pleomorphic adenoma
Intracranial involvement	Anterior skull base and middle cranial fossa	Orbital apex with extension to the anterior and middle cranial fossae
Surgical procedure	Orbitocranial resection with en bloc removal of orbital contents, involved dura, and affected orbital walls	Orbitocranial resection with removal of involved orbital walls and affected dura
Reconstruction	Temporalis fascia flap and split calvarial bone graft reconstruction	Temporalis fascia repair and split calvarial bone graft reconstruction
Margin status	Negative	Negative
Adjuvant treatment	Chemoradiotherapy (60 Gy/30 fractions + cisplatin-based chemotherapy)	Cisplatin-based chemoradiotherapy
Major postoperative complications	None	None
Follow-up duration	69-months	66-months
Disease status at last follow-up	Alive without evidence of disease recurrence	Alive without evidence of disease recurrence

## Conclusions

The orbitocranial approach, combined with postoperative chemoradiotherapy, may represent a feasible treatment option for selected patients with lacrimal gland malignant tumors with intracranial extension. In the present series, satisfactory local control and long-term survival were achieved following multidisciplinary management and orbital reconstruction. Careful preoperative evaluation, appropriate skull-base exposure, and long-term surveillance remain important components of treatment. Further studies with larger patient cohorts are required to better define the role of this approach in the management of these rare malignancies.

## ORCID ID

Yan Sui: 0009-0000-5456-1063

Si-Yi Yang: 0009-0009-4837-1496

Wen-Fu Lu: 0009-0005-0482-0721

## Funding

This work was supported by the Shenzhen Natural Science Foundation (Grant nº JCYJ20250604180831041 and JCYJ20220530142413031), the Translational Medicine Centre of The University of Hong Kong-Shenzhen Hospital supported by the Shenzhen-Hong Kong Cooperation Zone for Technology and Innovation (Grant nº HZQSWS-KCCYB-2024055).

## Data availability statement

The authors declare that all data are available in repository.

## Declaration of competing interest

The authors declare no conflicts of interest.

## References

[1] Aryasit O., Amornthatree P., Tsutsumi W.D. (2023). Clinical characteristics, radiologic features, and histopathology of biopsied lacrimal gland tumors. Sci Rep..

[2] Andreoli M.T., Aakalu V., Setabutr P. (2015). Epidemiological trends in malignant lacrimal gland tumors. Otolaryngol Head Neck Surg..

[3] Esmaeli B., Yin V.T., Hanna E.Y. (2016). Eye-sparing multidisciplinary approach for the management of lacrimal gland carcinoma. Head Neck..

[4] Hung J.Y., Wei Y.H., Huang C.H., Chen L.W., Fuh C.S., Liao S.L. (2019). Survival outcomes of eye-sparing surgery for adenoid cystic carcinoma of lacrimal gland. Jpn J Ophthalmol..

[5] Han J., Kim Y.D., Woo K.I., Sobti D. (2018). Long-term outcomes of eye-sparing surgery for adenoid cystic carcinoma of lacrimal gland. Ophthalmic Plast Reconstr Surg..

[6] Lin Y.H., Huang S.M., Yap W.K. (2020). Outcomes in patients with lacrimal gland carcinoma treated with definitive radiotherapy or eye-sparing surgery followed by adjuvant radiotherapy. Radiat Oncol..

[7] Esmaeli B., Golio D., Kies M., DeMonte F. (2006). Surgical management of locally advanced adenoid cystic carcinoma of the lacrimal gland. Ophthalmic Plast Reconstr Surg..

[8] Venkitaraman R., Madhavan J., Ramachandran K., Abraham E., Rajan B. (2008). Primary adenoid cystic carcinoma presenting as an orbital apex tumor. Neuroophthalmology..

[9] Rapidis A.D., Liarikos S. (1998). Malignant orbital and orbitomaxillary tumors: surgical considerations. Orbit..

[10] Wilson K.F., Ward P.D., Spector M.E., Marentette L.J. (2011). Orbitocranial approach for treatment of adenoid cystic carcinoma of the lacrimal gland. Ann Otol Rhinol Laryngol..

[11] Andreasen S., Esmaeli B., Holstein S.L., Mikkelsen L.H., Rasmussen P.K., Heegaard S. (2017). An update on tumors of the lacrimal gland. Asia Pac J Ophthalmol (Phila)..

[12] Tse D.T., Benedetto P., Dubovy S., Schiffman J.C., Feuer W.J. (2006). Clinical analysis of the effect of intraarterial cytoreductive chemotherapy in the treatment of lacrimal gland adenoid cystic carcinoma. Am J Ophthalmol..

[13] Ma M., Ren T., Liu R. (2024). Recurrent lacrimal gland adenoid cystic carcinoma: a case report and literature review. Heliyon..

[14] Anjum S., Sen S., Chosdol K. (2022). Vascular Endothelial Growth Factor (VEGF) and hypoxia inducible factor-1 alpha (HIF-1a) in lacrimal gland adenoid cystic carcinoma: Correlation with clinical outcome. Ann Diagn Pathol..

[15] Liu R., Li J., Zhang X., Ge X., Ma J. (2023). Differences in clinical features and prognosis between orbit adenoid cystic carcinoma and adenocarcinoma: a study from the SEER 18 database. Tumori..

[16] Tom A., Bell D., Ford J.R. (2020). Malignant mixed tumor (Carcinoma Ex Pleomorphic Adenoma) of the lacrimal gland. Ophthalmic Plast Reconstr Surg..

[17] Terasaki M., Tokutomi T., Maruiwa H., Sugita Y., Harada H., Shigemori M. (2000). High-grade adenoid cystic carcinoma originating from the lacrimal gland. Brain Tumor Pathol..

[18] Lee J.I., Kim Y.Z., Lee E.H., Kim K.H. (2008). Skull base invasion of adenoid cystic carcinoma of the lacrimal gland: a case report. J Korean Neurosurg Soc..

[19] Rose G.E., Gore S.K., Plowman N.P. (2019). Cranio-orbital resection does not appear to improve survival of patients with lacrimal gland carcinoma. Ophthalmic Plast Reconstr Surg..

[20] Boari N., Spina A., Giudice L., Gorgoni F., Bailo M., Mortini P. (2018). Fronto-orbitozygomatic approach: functional and cosmetic outcomes in a series of 169 patients. J Neurosurg..

[21] Marsh J.L., Wise D.M., Smith M., Schwartz H. (1981). Lacrimal gland adenoid cystic carcinoma: intracranial and extracranial en bloc resection. Plast Reconstr Surg..

[22] Brown B., Banerjee A.D., Wadhwa R. (2011). When is posterolateral orbitotomy useful in a pterional craniotomy? A morphometric study. Skull Base..

